# Effects of CREG1 on Age-Associated Metabolic Phenotypes and Renal Senescence in Mice

**DOI:** 10.3390/ijms22031276

**Published:** 2021-01-28

**Authors:** Michihiro Hashimoto, Ayumi Goto, Yuki Endo, Masataka Sugimoto, Jun Ueda, Hitoshi Yamashita

**Affiliations:** 1 Division of Advanced Medical Science, Asahikawa Medical University, Asahikawa 078-8510, Hokkaido, Japan; junueda@asahikawa-med.ac.jp; 2 Department of Biomedical Sciences, College of Life and Health Sciences, Chubu University, Kasugai 487-8501, Aichi, Japan; a-goto@isc.chubu.ac.jp (A.G.); yendo@isc.chubu.ac.jp (Y.E.); 3 Research Institute, National Center for Geriatrics and Gerontology, Obu 474-8511, Aichi, Japan; msugimot@ncgg.go.jp

**Keywords:** CREG1, cellular senescence, renal dysfunction, brown adipocyte, age-related obesity

## Abstract

Cellular repressor of E1A-stimulated genes 1 (CREG1) is a secreted glycoprotein that accelerates p16-dependent cellular senescence in vitro. We recently reported the ability of CREG1 to stimulate brown adipogenesis using adipocyte P2-CREG1-transgenic (Tg) mice; however, little is known about the effect of CREG1 on aging-associated phenotypes. In this study, we investigated the effects of CREG1 on age-related obesity and renal dysfunction in Tg mice. Increased brown fat formation was detected in aged Tg mice, in which age-associated metabolic phenotypes such as body weight gain and increases in blood glucose were improved compared with those in wild-type (WT) mice. Blood CREG1 levels increased significantly in WT mice with age, whereas the age-related increase was suppressed, and its levels were reduced, in the livers and kidneys of Tg mice relative to those in WT mice at 25 months. Intriguingly, the mRNA levels of *Ink4a*, *Arf*, and senescence-associated secretory phenotype (SASP)-related genes and p38MAPK activity were significantly lowered in the aged kidneys of Tg mice, in which the morphological abnormalities of glomeruli as well as filtering function seen in WT kidneys were alleviated. These results suggest the involvement of CREG1 in kidney aging and its potential as a target for improving age-related renal dysfunction.

## 1. Introduction

Cellular repressor of E1A-stimulated genes 1 (CREG1), which is a secreted glycoprotein containing 220 amino acids, inhibits cell growth and enhances the differentiation of human embryonic teratocarcinoma NTERA-2 cells [1,2]. CREG1 binds the tumor suppressor pRb in vitro [1] and enhances p16^INK4a^-induced senescence by the transcriptional repression of cell cycle-regulated genes, such as cyclin A and cyclin B, in immortal Li-Fraumeni Syndrome fibroblasts, and osteosarcoma and fibrosarcoma cell lines [3]. Several groups have also demonstrated that CREG1 overexpression protects against cardiac hypertrophy and fibrosis in the mouse heart and inhibits hepatic steatosis in the mouse liver [4,5]. The loss of CREG1 leads to early embryonic death, as it is essential for early myocardial development, and CREG1 heterozygous mice are susceptible to diet-induced obesity (DIO) and insulin resistance [6]. These studies suggest a potential role of CREG1 in age-associated disorders in vivo.

Similar to the p19^ARF^ (p14^ARF^ in human)/p53 pathway, the p16^INK4a^/Rb pathway is a major tumor suppressor pathway and plays critical roles in the induction and maintenance of cell cycle arrest during cellular senescence [7,8]. It is known that senescent cells show a specific phenotype called the senescence-associated secretory phenotype (SASP) [9,10]. Studies using the genetic elimination of senescent cells have made great advances in understanding the molecular mechanisms of aging including the progression of age-related diseases [11,12,13]. Gu et al. also reported that p16^INK4a^ plays an important role in exacerbating acute tubular necrosis in acute kidney injury, and the progression of these kidney injuries is improved in p16-knockout mice [14]. These reports indicate that the aging-associated decline in tissue functions is, at least partly, attributed to an accumulation of senescent cells in a subset of organs. However, the role of CREG1 in aging-associated phenotypes is completely unknown.

Brown adipose tissue (BAT) is specialized for thermogenesis through the function of mitochondrial uncoupling protein 1 (UCP1), which contributes to maintaining body temperature in cold environments in mammals [15,16]. Because UCP1 dissipates energy substrates such as glucose and fatty acids to produce heat, the promotion of brown adipogenesis also contributes to the prevention of obesity. In fact, UCP1 deficiency increases susceptibility to DIO and its associated glucose intolerance and fatty liver with age [17]. Additionally, the discovery of inducible brown-like adipocytes (beige/brite cells) in white adipose tissue (WAT), the so-called browning of the tissue, has accelerated basic and clinical research on the stimulation of brown adipocyte formation and activity as a potential target in the treatment of obesity and metabolic syndrome [18,19]. Sugino et al. recently reported an important role of brown and beige adipocytes in preventing inflammation and crystal formation in the mouse kidney [20]. Regarding the role of CREG1 in BAT thermogenesis, we recently reported that CREG1 is able to stimulate brown adipogenesis and UCP1 expression in vitro [21]. We also demonstrated that the overexpression of CREG1 in adipose tissue stimulated brown adipocyte formation and ameliorated DIO in adipocyte P2 (aP2)-CREG1 transgenic (Tg) mice, in which the progression of fatty liver and the decrease in insulin sensitivity and glucose tolerance were also improved [22]. These studies suggest that CREG1 plays an important role in energy metabolism through the regulation of adipocyte function and contributes to the improvement of obesity and its related pathology.

In this study, we investigated the effects of CREG1 overexpression on age-related changes in pathophysiology by using aP2-CREG1-Tg mice. This is the first report showing the inhibitory effect of CREG1 on age-related obesity and its relation to cellular senescence in kidney and renal function in mice.

## 2. Results

### 2.1. Improvement of Age-Related Obesity in aP2-CREG1-Tg Mice

**Figure 1:**
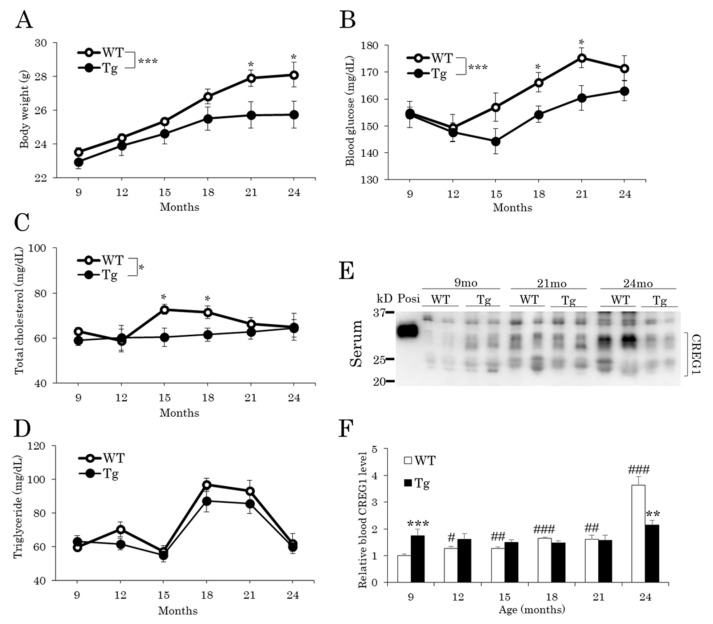
Changes in body weight and blood levels of glucose, lipids, and CREG1 in aged mice. (**A**) Body weight. (**B**) Fed glucose levels. (**C**) Total cholesterol levels. (**D**) Triglyceride levels. (**E**) Western blotting of CREG1 in serum. Positive control (Posi): mCREG1-MH [21]. A representative image is shown. (**F**) Relative CREG1 level in (**E**). Data are means ± SEM (*n* = 13 for *A* and *B*, *n* = 8 for (**C**–**F**) * *p* < 0.05, ** *p* < 0.01, *** *p* < 0.001 vs. wild type (WT) at the same time point; ^#^ *p* < 0.05, ^##^ *p* < 0.01, ^###^ *p* < 0.001 vs. 9 months in the same genotype.

The effect of CREG1 overexpression was investigated on aging-associated phenotypes in vivo. Age-related body weight gain was significantly suppressed in Tg mice compared to that in wild-type (WT) mice (Figure 1A) under normal chow diet conditions. We then analyzed the changes in metabolic parameters in the blood with aging and found that the increase in the blood glucose level with age was suppressed in Tg mice compared with that in WT mice (Figure 1B). In addition, the total blood cholesterol concentration was decreased in Tg mice at 15 and 18 months of age (Figure 1C), while there was no change in blood triglyceride concentration between the genotypes (Figure 1D). We then investigated how blood CREG1 levels changed with age. The serum CREG1 level in Tg mice was higher (1.75-fold) than in WT mice at 9 months of age (Figure 1E,F), consistent with our previous result found using 5-month-old mice [22]. Unexpectedly, however, the difference in serum CREG1 levels between genotypes was not found in 12-month-old to 21-month-old mice. By contrast, the CREG1 level was significantly higher in WT mice relative to that in Tg mice, which was due to its remarkable increase in WT mice with age (1.27-fold at 12 months and 3.64-fold at 24 months compared with 9 months). An age-related increase in blood CREG1 protein was not observed in Tg mice (Figure 1E,F).

### 2.2. Effect of Age on CREG1 Expression in Various Tissues of aP2-CREG1-Tg Mice

**Figure 2:**
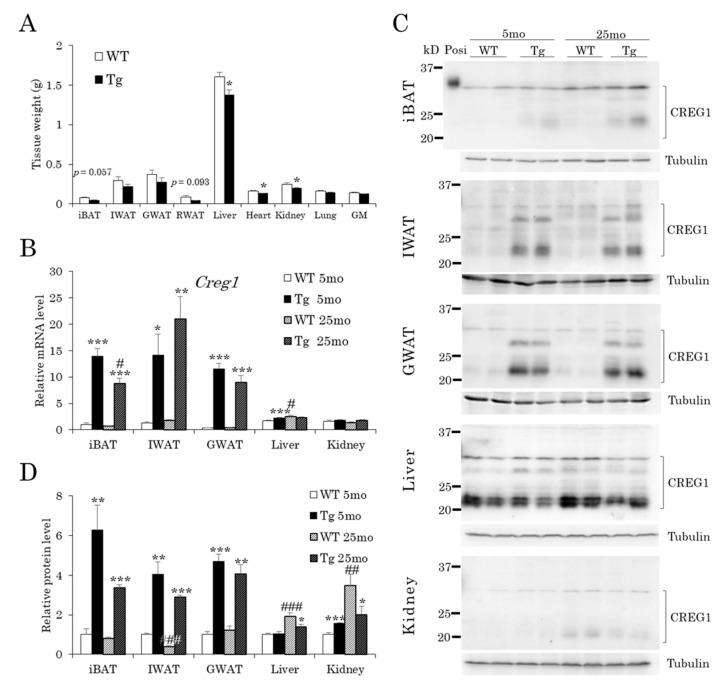
CREG1 expression levels in various tissues at 5 and 25 months. (**A**) Tissue weights. (**B**) mRNA expression of *Creg1.* (**C**) Western blotting for CREG1. (**D**) Relative protein level of CREG1 in (**C**). Positive control (Posi): mCREG1-MH; Tubulin: 55 kD. CREG1 levels were normalized to tubulin levels. Representative images are shown. iBAT: interscapular brown adipose tissue, IWAT: inguinal white adipose tissue, GWAT: gonadal white adipose tissue, RWAT: retroperitoneal white adipose tissue, GM: gastrocnemius muscle. Data are means ± SEM (*n* = 4–5). * *p* < 0.05, ** *p* < 0.01, *** *p* < 0.001 vs. WT at the same time point; ^#^ *p* < 0.05, ^##^ *p* < 0.01, ^###^ *p* < 0.001 vs. (**B**–**D**) 5 months for the same genotype.

Because serum CREG1 levels were lower in Tg mice than in WT mice in old age (Figure 1E,F), we examined CREG1 expression in several organs of 25-month-old mice (Figure 2A). The adipose tissues tended to be smaller, and the weights of the liver, heart, and kidney were significantly lower in Tg mice than in WT mice (Figure 2A).

We then measured the mRNA and protein levels of CREG1 in iBAT (interscapular brown adipose tissue), IWAT (inguinal white adipose tissue), GWAT (gonadal white adipose tissue), liver, and kidney of 5-month-old mice and aged mice. As expected, *Creg1* mRNA levels were 12–20-fold higher in each adipose tissue in young Tg mice, and this augmented expression was maintained in aged Tg mice compared to that in their WT littermates (Figure 2B). The mRNA expression of *Creg1* in the liver, the main tissue responsible for CREG1 expression at a steady-state level, was increased 1.4-fold in aged WT mice relative to young WT mice (Figure 2B). On the other hand, there was no difference in liver *Creg1* mRNA levels between the young and aged Tg mice, although the mRNA level was slightly but significantly higher in Tg mice than in WT mice at a young age (Figure 2B). No difference in *Creg1* mRNA levels was found between the age groups and genotypes in the kidney (Figure 2B). Consistent with the *Creg1* mRNA expression, the Western blotting of CREG1 showed a significant increase in protein levels in the adipose tissues of Tg mice (Figure 2C,D). The CREG1 level in WT livers was increased approximately two-fold in aged mice compared to young mice, whereas an age-related increase was not detected in Tg livers (Figure 2C,D). A similar pattern of CREG1 expression was detected in the kidney. Namely, a significant increase in the CREG1 level (3.5-fold) was found in aged kidneys relative to young kidneys in WT animals; however, this was not the case in Tg animals (Figure 2C,D). These results indicate that CREG1 levels increased with age in non-adipose tissues, including the liver, in WT mice, but their age-related increase was suppressed in aP2-CREG1-Tg mice, which may explain the difference in serum CREG1 levels between WT and Tg mice at 24 months (Figure 1E,F).

### 2.3. Stimulation of Browning and Improvement of Kidney Pathophysiology in Aged aP2-CREG1-Tg Mice

**Figure 3:**
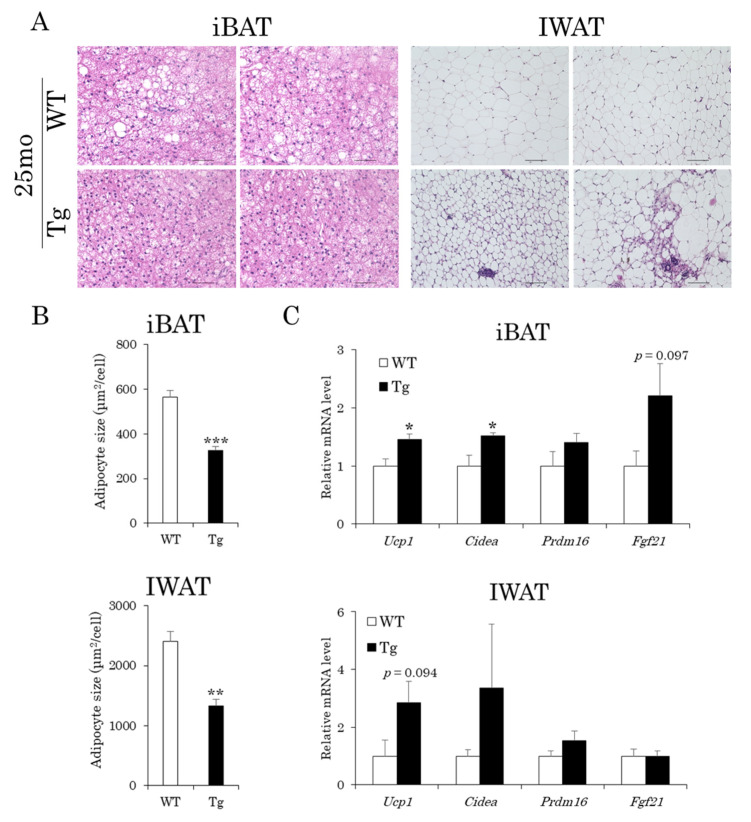
Browning is promoted in the adipose tissue of aged aP2-CREG1-Tg mice. (**A**) Histological analyses of iBAT and IWAT. Representative images of H&E staining are shown. Scale bar: 50 µm for iBAT, 100 µm for IWAT. (**B**) Adipocyte size in iBAT and IWAT. (**C**) mRNA expression of brown/beige fat-selective genes (*Ucp1, Cidea, Prdm16: PR domain containing 16,* and *Fgf21)* in iBAT and IWAT. Data are means ± SEM (*n* = 4). * *p* < 0.05, ** *p* < 0.01, *** *p* < 0.001 vs. WT.

We performed histological analysis of the adipose tissues in aged aP2-CREG1-Tg mice, as browning was promoted in young Tg mice [22]. Hematoxylin and eosin (H&E) staining revealed a decrease in lipid droplets in the iBAT and an increase in beige adipocytes in the IWAT of Tg mice compared to those of WT mice (Figure 3A). In fact, average cell sizes were reduced in the iBAT and IWAT of Tg mice (from 566 to 327 μm^2^ and from 2401 to 1328 μm^2^, respectively; Figure 3B). In addition, the mRNA levels of brown/beige fat-selective genes including *Ucp1*, *cell death-inducing DFFA-like effector a* (*Cidea*) and *fibroblast growth factor 21* (*Fgf21*, a regulator of brown/beige adipogenesis [19]) were increased in the iBAT of Tg mice compared with those of WT mice (Figure 3C). Similar trends in their mRNA expression except for *Fgf21* were detected in the IWAT of Tg mice compared with that of WT mice (Figure 3C). These results indicate that even in aged mice, browning is promoted in adipose tissues, in which the CREG1 protein levels are constantly high.

**Figure 4:**
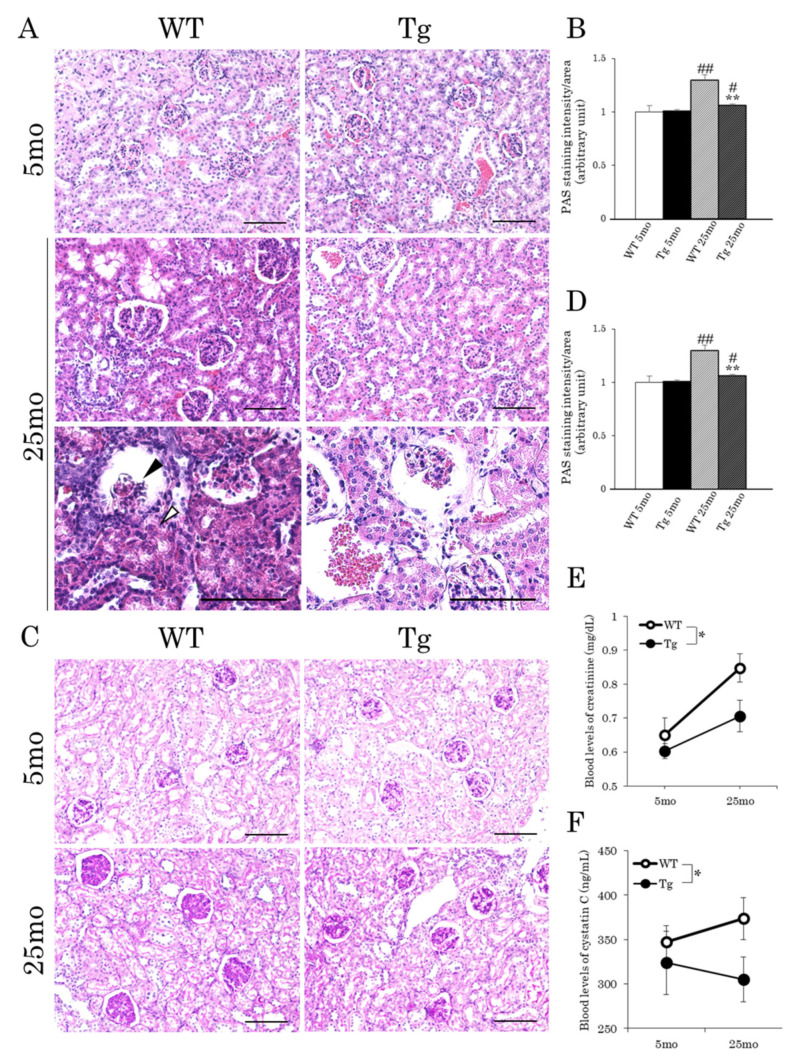
Age-related changes in kidney morphology and serum creatinine and cystatin C levels in aP2-CREG1-Tg mice. (**A**) H&E staining in kidney. The black arrow and white arrow indicate atrophied glomeruli and tubules, respectively. (**B**) Mean area of glomeruli (*n* = 4–5). (**C**) Periodic acid–Schiff (PAS) staining in kidney. (**D**) PAS staining intensity of glomeruli (*n* = 4–5). (**E**) Creatinine change in serum. (**F**) Cystatin C change in serum. (**A**,**C**) Scale bars, 100 µm. Representative images are shown. Data are means ± SEM (*n* = 4–5). * *p* < 0.05, ** *p* < 0.01 vs. WT at the same time point; ^#^ *p* < 0.05, ^##^ *p* < 0.01 vs. 5 months for the same genotype.

We next performed histological analysis of the kidney because it was recently reported that the promotion of browning prevented the formation of renal crystals and contributed to the improvement of renal function [20]. Glomerulosclerosis and tubular atrophy are known characteristics of the aging kidney [23,24]. Glomerular hypertrophy occurs in the early stages of diabetic nephropathy [25]. As shown in Figure 4A,B, hypertrophy of the glomeruli was observed in the kidneys of aged mice but not in those of young mice in both genotypes, whereas this aging-associated morphological change was moderated in the kidneys of Tg mice compared with that of WT mice. Atrophied glomeruli and renal tubules were also found in some areas of the aged WT kidneys (Figure 4A,B). In the process of kidney fibrosis, several types of collagen, glycosaminoglycans, glycoproteins, and polysaccharides accumulate in the renal interstitial space [26]. Periodic acid–Schiff (PAS) staining of the kidneys showed that the accumulation of mucus polysaccharides in aged glomeruli in WT mice was markedly reduced in Tg mice (Figure 4C,D). Indeed, the blood creatinine level was significantly lower in Tg mice than in WT mice at 25 months, while there was no difference at 5 months (Figure 4E). Similarly, the blood levels of cystatin C, a marker for kidney injury [27], tended to decrease in Tg mice compared to those in WT mice at 25 months, but not at 5 months (Figure 4F). These results suggest that the deterioration of renal filtration function with aging was improved in Tg mice.

### 2.4. Inhibition of Renal Senescence in Aged aP2-CREG1-Tg Mice

**Figure 5:**
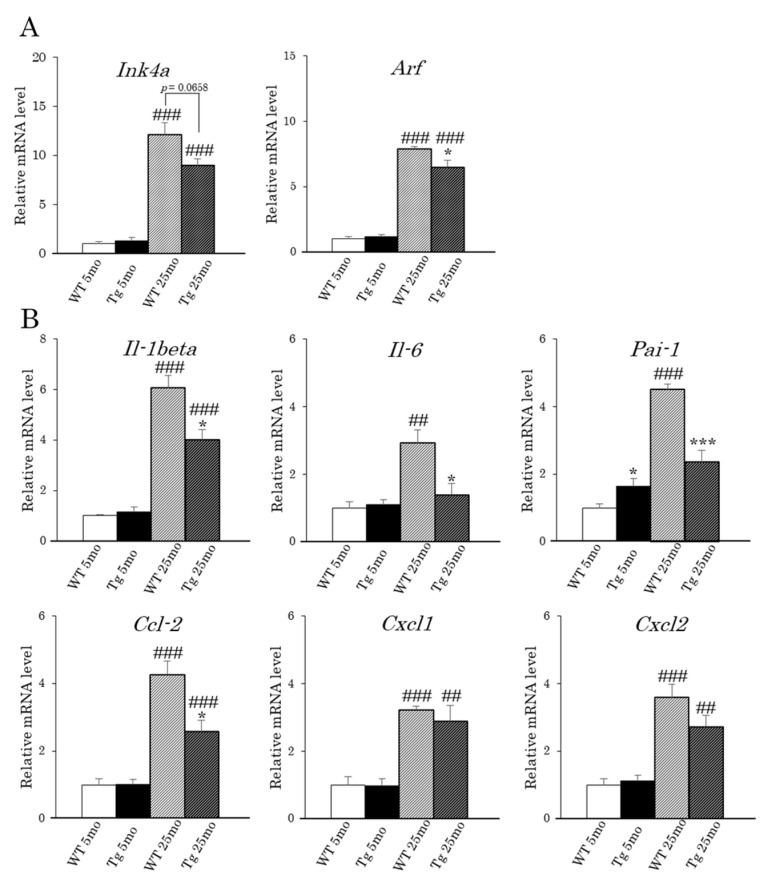
mRNA expression of cellular senescence-related genes in the kidney of aP2-CREG1-Tg mice. (**A**) mRNA levels of *Ink4a* and *Arf*. (**B**) mRNA levels of SASP-related genes (*Il-1beta, Il-6, Pai-1, Ccl-2, Cxcl1,* and *Cxcl2*). Total RNA isolated from kidneys was analyzed by real-time PCR. Data are means ± SEM (*n* = 4–5). * *p* < 0.05, *** *p* < 0.001 vs. WT at the same time point; ^##^ *p* < 0.01, ^###^ *p* < 0.001 vs. 5 months for the same genotype.

**Figure 6:**
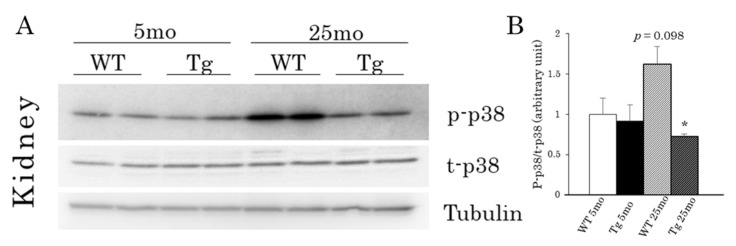
p38MAPK phosphorylation in kidneys of aP2-CREG1-Tg mice. (**A**) Western blotting of phosphorylated p38MAPK (p-p38), total-p38MAPK (t-p38) and tubulin. Tubulin was used as a loading control. (**B**) Relative phosphorylation level of p38MAPK in A; the phosphorylated level was normalized to total-p38MAPK. Data are means ± SEM (*n* = 4–5). * *p* < 0.05 vs. WT at 25 months.

To investigate the mechanism underlying the improvement of age-related renal dysfunction in Tg mice, we examined the expression levels of *Ink4a* and *Arf*, both of which are thought to be critical genes in the induction of senescence [7,8]. The *Ink4a* and *Arf* mRNA levels in the kidney were significantly higher in the 25-month-old aging groups than in the young groups for both genotypes (12-fold in WT and 6.9-fold in Tg, and 7.9-fold in WT and 5.5-fold in Tg, respectively), whereas these mRNA levels were lower in Tg mice than in WT mice at 25 months (Figure 5A,B). We then analyzed the expression of SASP-related cytokines (*Il-1beta, IL-6, Pai-1, Ccl-2, Cxcl1*, and *Cxcl2*). These cytokines are also involved in the inflammatory response in various diseases, including nephrosclerosis. As shown in Figure 5B, these mRNA levels were markedly increased in aged WT and Tg mice compared to those in their young littermates. However, the increased mRNA expression of *Il-1beta, Il-6, Pai-1,* and *Ccl-2,* but not *Cxcl1* and *Cxcl2,* in the aged animals was significantly smaller in Tg mice compared to that in WT mice (Figure 5B). We finally examined the phosphorylation level of p38MAPK, since its signaling pathway directly controls senescence and *Ink4a* expression in endothelial progenitor cells [28,29]. As shown in Figure 6, the phosphorylation level of p38MAPK was significantly suppressed in aged Tg mice compared with that in WT littermates, although there was no difference in the phosphorylation levels between the young WT and Tg mice.

## 3. Discussion

There is accumulating evidence that CREG1 is a unique endocrine factor that plays important roles in the regulation of gene expression, cell growth and differentiation, and metabolism in mice. We previously demonstrated that CREG1 overexpression in adipose tissues enhanced BAT function and induced WAT browning in aP2-CREG1-Tg mice, which showed a phenotype resistant to DIO as compared with WT mice [22]. Glucose tolerance and lipid metabolism were also improved in Tg mice compared with those in WT mice under the high-fat-diet condition. These beneficial effects of CREG1 overexpression were evaluated in aged mice in this study because normal C57BL/6 mice gained a significant amount of weight with age even under standard dietary conditions, and glucose intolerance became prominent in aged animals [30]. As expected, age-related obesity as well as the elevation of blood glucose and lipid levels in WT mice was improved in Tg mice. Although BAT function is attenuated in aged animals [31], the deterioration was suppressed in aged Tg mice relative to WT mice. It is probable that the adipose-specific induction of CREG1 contributed to the improvement of age-related metabolic phenotypes by stimulating brown/beige adipocyte formation in iBAT and IWAT in Tg mice, although serum CREG1 levels were unexpectedly lower in Tg mice than in WT mice at 24 months. The promotion of brown/beige adipocyte formation was thought to be regulated by the autocrine/paracrine action of CREG1 in adipose tissues as reported in our previous studies [21,22]. The reduction in serum CREG1 levels in Tg mice may reflect a decrease in CREG1 protein expression in the liver, the major organ for producing CREG1, and suggest a negative regulation (e.g., in the translation step) in response to the successive increase in blood CREG1 supplied from the adipose tissues in the mutant mice. In contrast to Tg mice, we found that serum CREG1 levels increased in normal WT mice with age. It is presently unknown why the age-related increase occurred in WT mice. This increase in serum CREG1 level may indicate the progress of resistance to CREG1, such as insulin resistance, with age. However, the mechanism remains to be verified. Likewise, it is uncertain whether there is a gender difference in the effects of CREG1 on aging-associated phenotypes. We used female mice in this study, as several studies, including our previous aging study, reported that females were more dependent on UCP1 thermogenesis than males for maintaining body temperature in the cold and avoiding obesity with age [17,32]. In the future, it is expected that an aging analysis using male Tg mice will be conducted to reveal more details regarding the physiological functions of CREG1 in aging-associated phenotypes.

A previous study reported that senescent cells accumulate in many tissues, including the kidney, with age [33]. These senescent cells may cause renal dysfunction and nephrosclerosis in the aging kidney [23,24]. In fact, the clearance of senescent cells in the kidney delays age-related disorders in genetically modified old mice [11,12,13,34,35]. Because CREG1 accelerates p16-dependent cellular senescence in vitro [3], understanding the role of CREG1 is important in understanding the pathogenic mechanisms of age-related disorders in the kidney. Intriguingly, the age-related dysfunctional morphology such as glomerular and tubular atrophy seen in WT mice was improved in Tg mice, in which CREG1 protein levels and the mRNA levels of *Ink4a* and *Arf* were lowered in the kidneys, suggesting that cellular senescence was suppressed in the aging kidneys of Tg mice. This idea is supported by the finding that the mRNA levels of SASP-related cytokines such as *Il-1beta*, *Il-6*, *Pai-1*, and *Ccl-2* were significantly decreased in the aged kidneys of Tg mice compared with those of WT mice. Analysis of mucus polysaccharides suggested the suppression of kidney fibrosis in Tg mice relative to WT mice. Moreover, serum levels of creatinine and cystatin C were reduced in aged Tg mice compared with those in WT mice. Creatinine is a metabolite of creatinine phosphate, a source of energy for muscles, which is normally filtered by the kidneys and excreted in the urine. Similarly, cystatin C detects acute kidney injury early in cecal ligation and puncture-induced mice, and the combination of serum creatinine and serum cystatin C acts as a better biomarker for renal filtration [27]. Together, our results indicate that the filtering function of the kidney in Tg mice is better than that in WT mice in old age. Furthermore, the stimulation of brown/beige adipocyte formation might have indirectly affected the improvement of renal function in aged Tg mice because these thermogenic adipocytes reduce inflammation in the kidney and contribute to the prevention of renal crystal formation [20]. Contradictory to what is observed in the kidney, the high expression of CREG1 may not enhance cellular senescence in the adipose tissues of Tg mice, as was suggested by the morphological and gene expression analyses. For example, the sizes of the lipid droplets and cells in iBAT usually become larger in normal mice with age (called whitening), which is often associated with a decrease in *Ucp1* expression. On the other hand, these age-related changes were suppressed in the aged iBAT of Tg mice compared with those in WT mice. These results suggest that CREG1 function is distinct among tissues (i.e., iBAT and kidneys) or cell types. Because CREG1 can affect the cell cycle and stimulate cell differentiation, this ability could act on the maintenance of tissue such as adipose tissue composed of mainly differentiated adipocytes.

Finally, the phosphorylation level of p38MAPK was found to be reduced in the aged kidney of Tg mice relative to that of WT mice. p38MAPK participates in a signaling cascade that controls cellular responses to cytokines and stress. A previous study reported that p38MAPK is activated by many stimuli, including IL-1 and TNF-α, which play a critical role in renal pathophysiology [36]. The inhibition of p38MAPK signaling using SB203580, a selective p38MAPK inhibitor, improved renal disease in a mouse model of systemic lupus nephritis [37]. Experiments using a rat model of renal artery stenosis also revealed that the progression of renal atrophy and fibrogenesis was improved by the inhibition of the downstream targets of the p38MAPK pathway [38]. Additionally, it is well established that p38MAPK mediates the stress signal to induce senescence-associated cell cycle arrest as well as SASP [39]. However, p38MAPK could also be activated by inflammatory cytokines as a consequence of SASP. Therefore, it is reasonable to assume that the phosphorylation of p38MAPK reflects the changes in both the senescence-inducing signal and SASP activity. Considering these findings, it is plausible that the reduced level of CREG1 acted directly in inhibiting cellular senescence in the kidney, and it resulted in a decrease in SASP stress and p38MAPK activity in the aged kidney, contributing to the maintenance of renal function in Tg mice compared with that in WT mice. In that sense, the decrease in the CREG1 level appeared to be beneficial for kidney aging, although this may not be compatible with BAT activation.

Thus, an indirect effect of CREG1 through thermogenic adipocytes and a direct effect of CREG1 on cellular senescence in the kidney seem to be involved in the mechanism underlying the improvement of renal pathophysiology in aging aP2-CREG1-Tg mice. Controlling the protein levels of CREG1 in vivo may lead to the development of new therapies for age-related kidney disease; however, the regulatory mechanism of CREG1 production and precise role of CREG1 in the kidney remain to be addressed in further in vivo studies by controlling the expression of CREG1 in a kidney-specific manner.

## 4. Materials and Methods

### 4.1. Animals

For the generation of aP2-CREG1-Tg mice, the complete mouse CREG1 cDNA was cloned 3′ to the 5.66-kb aP2 promoter, and the bovine growth hormone polyadenylation site was inserted 3′ to the cDNA. The aP2-mCREG1 DNA was microinjected into BDF1 embryos, and the Tg founders were backcrossed to the C57BL/6J mice at least five times. [22]. In the aging experiment, WT and Tg mice (line 52) were provided with standard chow (CE-2; CLEA Japan, Inc., Sizuoka, Japan) and tap water. These mice were maintained at 23 °C. Body weight was measured from 9 to 24 months of age. After 9 months of age, blood samples were collected up to 24 months of age and stored at −30 °C. At 25 months of age, tissue samples were collected and stored at −80 °C or fixed in 4% paraformaldehyde in PBS. Unless otherwise noted, female mice were used in these aging experiments. The experiments in this study were performed in strict accordance with the recommendations in the Fundamental Guidelines for Proper Conduct of Animal Experiment and Related Activities in Academic Research Institutions under the jurisdiction of the Ministry of Education, Culture, Sports, Science, and Technology, Japan. The protocol was approved by the Institutional Animal Care and Use Committee of Chubu University (#2610041, 17 March 2014) and the Institutional Animal Care and Use Committee at Asahikawa Medical University (#19156, 20 August 2019; #20090, 1 June 2020; #20090-2, 21 December).

### 4.2. Blood Glucose Level Measurement

Blood glucose levels were determined using a glucometer (NovoAssist Plus, Novo Nordisk, Tokyo, Japan) under free-feeding conditions.

### 4.3. Lipid Measurement

Serum total cholesterol and triglyceride levels were measured using T-Cho E-test and TG E-test kits (Wako Pure Chemical, Osaka, Japan), respectively. The protocol given in the attached instruction manual was followed.

### 4.4. Gene-Expression Analysis

Total RNA was extracted using TRIzol reagent (Thermo Fisher Scientific) according to the manufacturer’s protocol. The total RNA was reverse-transcribed using a High-Capacity cDNA Reverse Transcription Kit (Thermo Fisher Scientific) as per the manufacturer’s instructions. Real-time RT-PCR analysis was performed using a Light-Cycler^®^ or CFX Connect, and THUNDERBIRD^TM^ SYBR^®^ qPCR Mix (TOYOBO, Osaka, Japan), THUNDERBIRD^TM^ Next SYBR^®^ qPCR Mix (TOYOBO, Osaka, Japan), or FastStart DNA Master^Plus^ Sybr Green I (Roche, Basel, Switzerland) to quantify mRNA expression levels. All gene-expression data were normalized relative to 36B4 levels. The following oligonucleotide primer sets were used to perform PCR: for *36b4*, 5′-TCATCCAGCAGGTGTTTGACA-3′ (sense) and 5′-CCCATTGATGATGGAGTGTGG-3′ (antisense); *Creg1*, 5′-GACCTGCAGGAAAATCCAGA-3′ (sense) and 5′-AACAAACAGCGAATCCCTTG-3′ (antisense); *Ucp1*, 5′-GTGAAGGTCAGAATGCAAGC-3′ (sense) and 5′-AGGGCCCCCTTCATGAGGTC-3′ (antisense); *Cidea*, 5′-ATCACAACTGGCCTGGTTACG-3′ (sense) and 5′-TACTACCCGGTGTCCATTTCT-3′ (antisense); *Prdm16*, 5′-GACATTCCAATCCCACCAGA-3′ (sense) and 5′-CACCTCTGTATCCGTCAGCA-3′ (antisense); *Fgf21*, 5′-GTGTCAAAGCCTCTAGGTTTCTT-3′ (sense) and 5′-GGTACACATTGTAACCGTCCTC-3′ (antisense); *Arf*, 5′-GCCGCACCGGAATCCT-3′ (sense) and 5′-TTGAGCAGAAGAGCTGCTACGT-3′ (antisense); *Ink4a*, 5′-CCCAACGCCCCGAACT-3′ (sense) and 5′-GCAGAAGAGCTGCTACGTGAA-3′ (antisense); *Il-1beta*, 5′-GAATGCCACCTTTTGACAGTG-3′ (sense) and 5′-CTGGATGCTCTCATCAGGACA-3′ (antisense); *Il-6*, 5′-CAAGAAAGACAAAGCCAGAGTC-3′ (sense) and 5′-GAAATTGGGGTAGGAAGGAC-3′ (antisense); *Pai-1*, 5′-TCAGAGCAACAAGTTCAACTACACTGAG-3′ (sense) and 5′- CCCACTGTCAAGGCTCCATCACTTGCCCCA-3′ (antisense); *Ccl-2*, 5′-TAAAAACCTGGATCGGAACCAAA-3′ (sense) and 5′-GCATTAGCTTCAGATTTACGGGT-3′ (antisense); *Cxcl1*, 5′-ACTGCACCCAAACCGAAGTC-3′ (sense) and 5′-TGGGGACACCTTTTAGCATCTT-3′ (antisense); *Cxcl2*, 5′-TAAAAACCTGGATCGGAACCAAA-3′ (sense) and 5′-GCATTAGCTTCAGATTTACGGGT-3′ (antisense); *Igf2R*, 5′-GCACCAAGATGAAGCAGTCA-3′ (sense) and 5′-ACATCCGGTAGCTGTTGGTC-3′ (antisense); *Cathepsin B*, 5′-GGGGACGGCTGTAATGGTGG-3′ (sense) and 5′-CCCAGCCCAGGATGCGGATG-3′ (antisense) [40].

### 4.5. Protein Analysis

Protein levels were analyzed by Western blotting using total tissue lysates (50 µg) and serum samples (1 µL) as previously described [22]. Protein samples were separated using SDS-PAGE and transferred onto PVDF membranes (MilliporeSigma, Burlington, MA, USA), which were then incubated with specific antibodies against α-/β-tubulin (2148; Cell Signaling Technology, Danvers, MA, USA), CREG1 (C-17, sc-11728; Santa Cruz Biotechnology, Dallas, TX, USA), p38 MAPK (#9212; Cell Signaling, Danvers, MA, USA), and phospho-p38 MAPK (Thr180/Tyr182) (#9211; Cell Signaling). After incubation with secondary antibodies for 1 h at room temperature, specific immunoreactive signals were detected using the Immobilon Western Chemiluminescent Horseradish Peroxidase Substrate (MilliporeSigma). The mCREG1-MH recombinant protein was used in the Western blot analysis as a positive control to ensure that the specific antibody against CREG1 was functioning. Namely, a C-terminal His-tag-fused CREG1 expression vector (pcDNA-mCREG1-MH) was constructed by inserting mouse CREG1 cDNA into the pcDNA3.1/Myc-His vector. Cos7 cells were transfected with pcDNA-mCREG1-MH using polyethylenimine. mCREG1-MH protein secreted in the culture media was purified by ammonium precipitation, HisTrap HP affinity chromatography, and molecular exclusion chromatography with Superdex G-75 [21].

### 4.6. Histologic Analysis

Fixed tissues were embedded in paraffin wax, sectioned at a 6 µm thickness, and stained with H&E (Wako Pure Chemicals, Osaka, Japan). The sections were imaged using a BZ-X700 microscope (Keyence Corp., Osaka, Japan). The average area of adipocytes was calculated using the H&E staining images and the ImageJ software (National Institutes of Health, Bethesda, MD, USA). The average area of iBAT and IWAT cells was calculated from more than 2000 cells/mouse and 800 cells/mouse, respectively. The average glomerular area was calculated using randomly selected images × 20 fields (40–80 fields per mouse) and the ImageJ software. PAS staining and sections with a thickness of 4 µm were randomly selected for dewaxing and hydration, and then stained in PAS dye solution. Counterstaining was performed with Mayer’s hematoxylin as mentioned above. The mean intensity of PAS staining in glomeruli per mouse was calculated using randomly selected images × 20 fields (at least 60 glomeruli per mouse) and the ImageJ software.

### 4.7. Evaluation of Renal Function

Serum creatinine and cystatin C levels were measured to assess renal function. The serum creatinine and cystatin C levels were measured using a DetectX^®^ Serum Creatinine Detection Kit (ARBOR ASSAYS, Ann Arbor, MI, USA) and MOUSE CYSTATIN C ELISA (BioVendor, Brno, Czech Republic), respectively. The protocol in the attached instruction manual was followed.

### 4.8. Statistical Analysis

Data are expressed as mean ± SEM. Differences between the two groups in the mouse studies, including differences in body weight, blood glucose levels, and lipid measurements, were analyzed using repeated-measurements two-way ANOVA. Other statistical comparisons were performed using a two-tailed Student’s *t*-test. Values of *p* < 0.05 were considered statistically significant.

## Data Availability

The data that support the findings of this study are available from the corresponding author upon reasonable request.

## Abbreviations

aP2	Adipocyte P2
C57BL/6J	C57 black 6 Jackson
CIDEA	Cell death-inducing DFFA-like effector A
CREG1	Cellular repressor of adenovirus early region 1A-stimulated genes 1
DIO	Diet-induced obesity
FGF21	Fibroblast growth factor 21
GM	Gastrocnemius muscle
GWAT	Gonadal white adipose tissue
H&E	Hematoxylin and eosin
iBAT	Interscapular brown adipose tissue
IWAT	Inguinal white adipose tissue
PAS	Periodic acid–Schiff
pRb	Retinoblastoma tumor suppressor protein
PRDM16	PR domain containing 16
RWAT	Retroperitoneal white adipose tissue
SASP	Senescence-associated secretory phenotype
Tg	Transgenic
UCP1	Uncoupling protein 1
WT	Wild type
